# 

**Published:** 1893-04-29

**Authors:** 


### CONTENTS.

Equal Service, Equal Honour . . . . . . . . 65
Around the Hospitals . . . . . . . . . . 66
Medical History from the Earliest Times. By E. T. Withington, M.A., M.B Oxon. LII.—Medical Practice in the Sixteenth Century . . . . . . . . . 67
Contemporary Theory and Research . . . . . . 68
Great Achievements Under Suffering, I. Prof. H. Fawcett 69
Gleanings in Science and Medicine . . . . . . 70
Annotations.—Mr. Balfour and "Research"—Laziness as a Cure—The Afternoon Nap . . . . . . . . . . 71
Notes and News . . . . . . . . . . . 72
Scraps and Gleanings . . . . . . . . . . 80
The Book World of Medicine and Science . . . . viii
The Hospital Clinic—The Leeds General Infirmary.—Treatment of Simple Fractures of the Femur . . . . 73
Central London Throat Hospital.—The Treatment of Rhinitis 74
The Practitioners' Bookshelf.—Vaccination Eruptions . . 76
The Institutional Workshop—
Within the Hospitals.—Children's Hospitals in East London—The East London Hospital for Children . . . . . 77
Practical Departments.—Invalid Chairs, II. . . . . . 79
The Redcliffe Infirmary, Oxford . . . . . . . 79
Hospital Opinion.—The Proposed Central Hospitals Board for London . . . . . . . . . . . . . 80
Festival Dinners.—The North London Hospital for Consumption 80
The Student of Medicine.—Appointments, Vacancies .

EXTRA SUPPLEMENT.—THE NURSING MIRROR.

News from the Nursing World.—The Nurse Dolls—Moderate and Immoderate Exercise—Novel Scholarships—Nurses as Scrubbers—Queen's Nurses at Ryde—Training for Village Nurses—Unwelcome Retrenchment—A New Nurses' Home — Twenty-eight Years After — Stirling — Misapplied Economy—Dublin—More Nurses for India—Short Items—The Disabled Nurse . . . . . . . . xli

Sanitation for Nurses. By A Sanitary Inspector. III.—Notification in Foreign Countries . . . . . . . xliii

A German Hospital By an English Patient . . . xliv

Notes and Queries . . . . . . . . . . xliv

The Jubilee Institute at Taunton . . . . . . xlv

Nursing Subjects at Chicago . . . . . . . xlvi

The Vaterländische Frauenverein . . . . . . . xlvi

Up-Country Nursing Association for Europeans in India.—For Reading to the Sick.—The Prescription. II. xlvii

Where to Go.—Wants and Workers.—Presentation . xlvii

The Infectiousness of Consumption . . . . . xlviii

Her Better Self.—Maternal Sanitation . . . . xlviii

The Book World for Women and Nurses . . . . xlix

